# Supplementary material for: Proscillaridin A Sensitizes Human Colon Cancer Cells to TRAIL-Induced Cell Death
Source: Int J Mol Sci. 2022 Jun 23;23(13):6973. doi: 10.3390/ijms23136973 (PMC9266755; doi:10.3390/ijms23136973)
Supplement: Supplementary file 1 [file ijms-23-06973-s001.zip › ijms-1772019-supplementary.pdf]

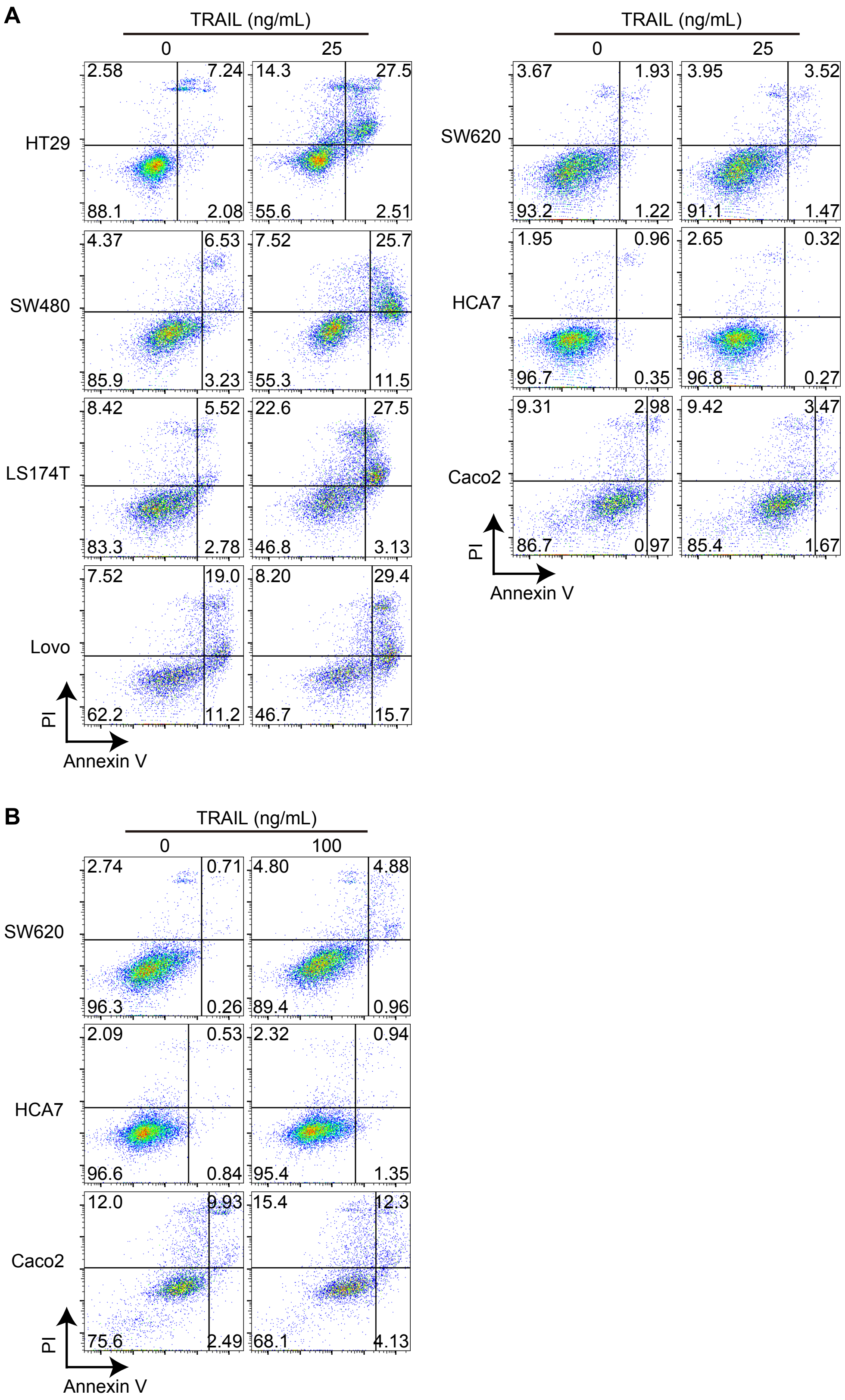

Supplementary Figure S1. TRAIL-induced cell death in various colon cancer cell lines. Cells were treated with TRAIL at the indicated concentration for 16 h. Representative FACS plots from triplicates are shown.

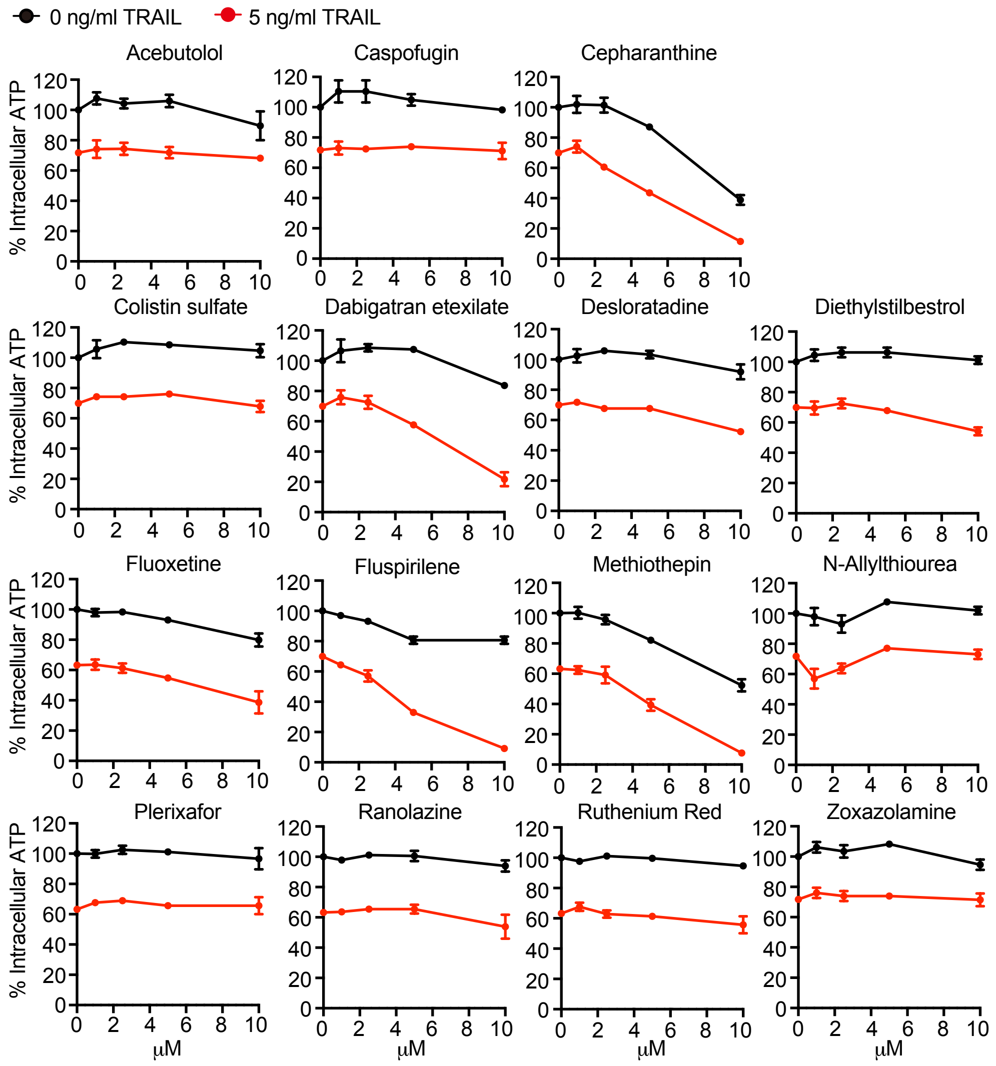

Supplementary Figure S2. Validation of the hit compounds for their TRAIL-sensitizing effect. HT29 cells were pretreated with each compound at the indicated concentration for 24 h and subsequently stimulated with TRAIL for 16 h in the presence of each compound. The intracellular ATP level was determined by CellTiter-Glo luminescent cell viability assay. Results are mean ± SD of triplicate samples.

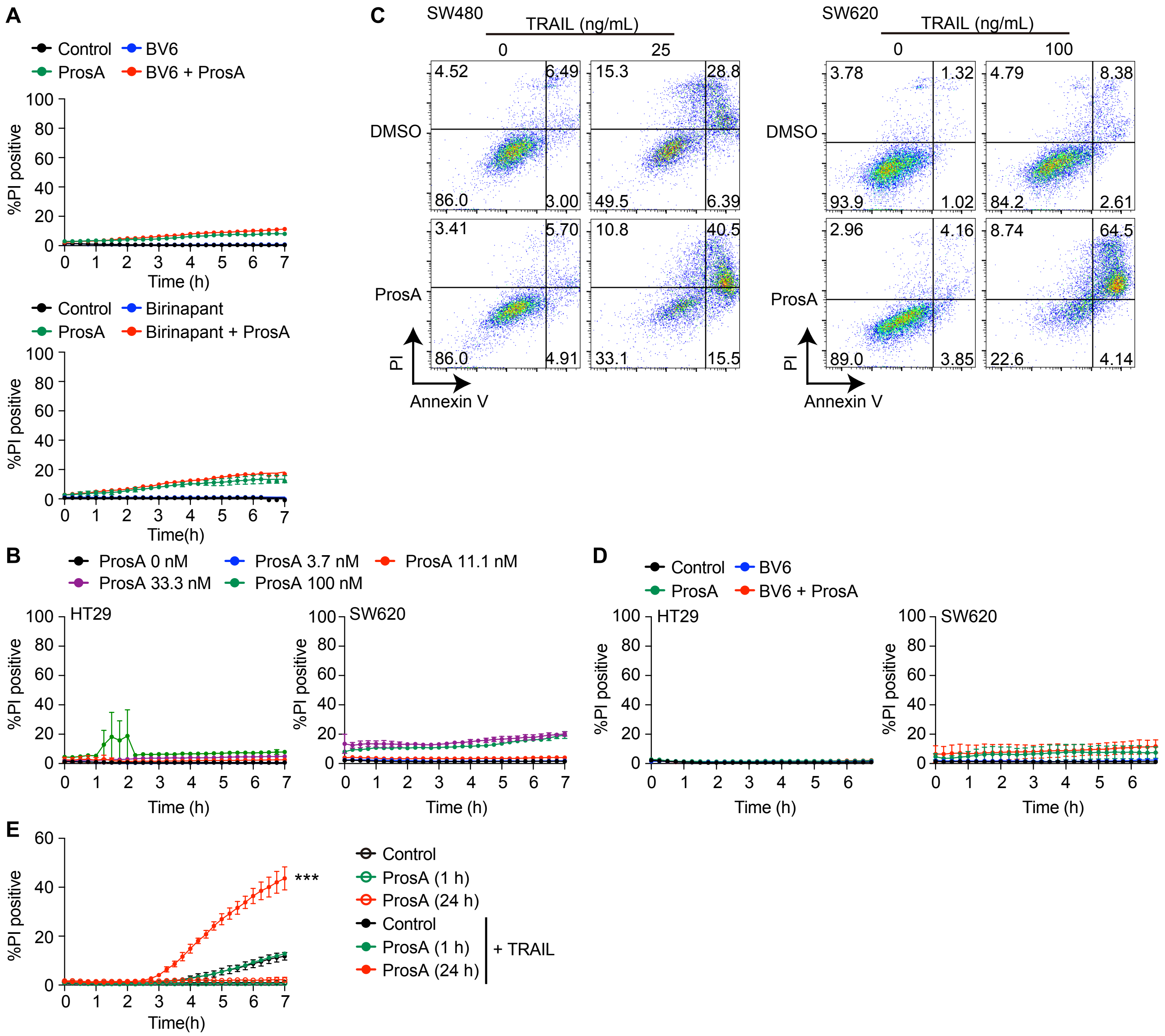

Supplementary Figure S3. Prosillaridin A enhanced TRAIL-induced apoptosis.
(**A, D**) HT29 cells were pretreated with 1 µM (**A**) or 11.1 nM (**D**) proscillaridin A (ProsA) for 24 h and subsequently treated with 0.5 µM SMAC mimetic (BV6 or birinapant) for 1 h. After that, the cells were monitored for the uptake of PI. Results obtained with TRAIL are shown in Figure 2B (**A**) or 2E (**D**). (**B**) HT29 and SW620 cells were pretreated with proscillaridin A (ProsA) for 24 h. After that, the cells were monitored for the uptake of PI. Results obtained with TRAIL are shown in Figure 2C. (**C**) SW480 and SW620 cells were pretreated with 11.1 nM proscillaridin A for 24 h and then treated with TRAIL for 16 h. Representative FACS plots from triplicates. (**E**) HT29 cells were pretreated with 11.1 nM ProsA for 1 or 24 h and then treated with 10 ng/ml TRAIL. The cells were monitored for the uptake of PI. Results are obtained in triplicate. Results are representatives of two independent experiments (**A**-**E**). Results are represented as the mean ± SD (**A, B, D, E**).

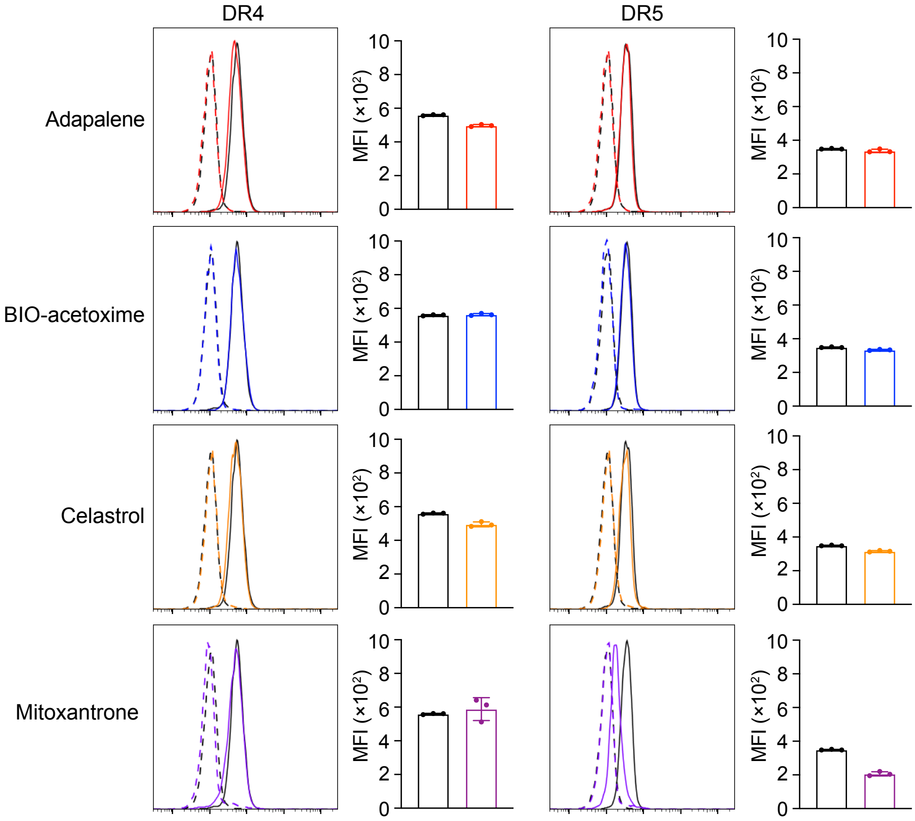

Supplementary Figure S4. Cell surface expression of TRAIL receptors after treatment with various compounds. HT29 cells were treated with 1 μM compounds for 24 h. Cell surface expression of DR4 and DR5 was determined by flow cytometer. Dotted lines indicate the cells stained without primary antibodies. Colored and black lines indicate the cells treated with and without compounds, respectively. Representative histograms from three independent experiments are shown. Bar-dot graphs indicate the mean fluorescence intensity (MFI). Results are represented as the mean ± SD of triplicate.

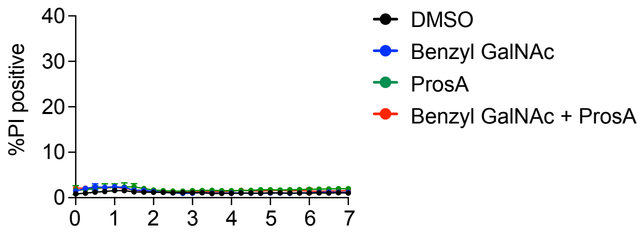

Supplementary Figure S5. Cell viability after co-treatment with proscillaridin A and benzyl-α-GalNAc. HT29 cells were pretreated with 11.1 nM proscillaridin A (ProsA) and 2 mM benzyl-α-GalNAC for 24 h. After that, the cells were monitored for the uptake of PI. Results obtained with TRAIL are shown in Figure 5B. Results are represented as the mean ± SD of triplicate. Results are representative of two independent experiments.
